# Bacteria Detected in both Urine and Open Wounds in Nursing Home Residents: a Pilot Study

**DOI:** 10.1128/mSphere.00463-19

**Published:** 2019-08-28

**Authors:** Josie Libertucci, Christine M. Bassis, Marco Cassone, Kristen Gibson, Bonnie Lansing, Lona Mody, Vincent B. Young, Jennifer Meddings

**Affiliations:** a Department of Internal Medicine, Infectious Diseases Division, University of Michigan Medical School, Ann Arbor, Michigan, USA; b Department of Internal Medicine, Division of Geriatric and Palliative Medicine, University of Michigan Medical School, Ann Arbor, Michigan, USA; c Center for Clinical Management Research, Veterans Affairs Ann Arbor Healthcare System, Ann Arbor, Michigan, USA; d Department of Medicine, Veterans Affairs Ann Arbor Healthcare System, Ann Arbor, Michigan, USA; e Department of Microbiology and Immunology, University of Michigan Medical School, Ann Arbor, Michigan, USA; f Department of Pediatrics and Communicable Diseases, Division of General Pediatrics University of Michigan Medical School, Ann Arbor, Michigan, USA; University of Nebraska Medical Center

**Keywords:** bacteriology, clinical microbiology, nursing home residents, urine microbiota, wound microbiota

## Abstract

Nursing home residents are at a greater risk of developing pressure injuries that develop into an open wound, which can become colonized with bacteria. Understanding the factors that influence microbial colonization of open wounds can lead to the prevention of infections. The relationship between bacteria found in urine and those in open wounds is currently unknown. To determine if bacterial species colonizing open wounds are also found in the urine, we conducted a pilot study with nursing home residents, comparing bacterial species present in the urine with those present in wounds between the umbilicus and mid-thigh. To identify microbial species that were present in both urine and open wound at one time point in one patient, standard clinical bacteriologic culture techniques followed by matrix-assisted laser desorption ionization–time of flight mass spectrometry (MALDI-TOF MS) were used, as well as 16S rRNA-encoding gene amplicon sequencing. We found some bacterial species detected in urine were also detected in open wounds in one individual at one time point, using both culture-dependent and -independent techniques. Bacterial species that were more often detected, using culture-dependent and -independent methods, at both sites included Enterococcus faecalis, Proteus mirabilis, Escherichia coli, and Providencia stuartii. This pilot study provides evidence that bacterial species identified within the urine can also be identified in open wounds in the same patient at one point in time. Further studies are needed to investigate if these species are of the same lineage and if the urinary microbiota are able to seed colonization of open wounds below the umbilicus.

**IMPORTANCE** Older adults, specifically those in nursing facilities, are more susceptible to developing chronic open nonhealing wounds. Chronic open nonhealing wounds severely impact an individual’s quality of life and can lead to other comorbidities, such as infection. Recent evidence suggests that the open wound bacterial community can influence wound healing and repair. It is important to understand all sources of open wound contamination to improve preventative infection measures and treatment protocols. In this pilot study, we investigated if bacterial species isolated from urine can also be isolated from open wounds located between the levels of the umbilicus and mid-thigh in the same patient at the same point in time. A growing body of evidence suggests that urine can harbor a microbial community, even in asymptomatic individuals, and older adults are more prone to urinary incontinence. This is the first study to investigate bacterial species concordance between these two anatomical sites. We found, using both culture-dependent and -independent methods, that the same bacterial species can colonize both the urine and wound in one patient at one point in time. Further studies are needed to investigate if these species are of the same lineage and if the urinary microbiota are able to seed colonization of these types of open wounds.

## Abstract (precis)

Older adults, specifically those in nursing facilities, are more susceptible to developing chronic open nonhealing wounds. Chronic open nonhealing wounds severely impact an individual’s quality of life and can lead to other comorbidities, such as infection. Recent evidence suggests that the open wound bacterial community can influence wound healing and repair. It is important to understand all sources of open wound contamination to improve preventative infection measures and treatment protocols. In this pilot study, we investigated if bacterial species isolated from urine can also be isolated from open wounds located between the levels of the umbilicus and mid-thigh in the same patient at the same point in time. A growing body of evidence suggests that urine can harbor a microbial community, even in asymptomatic individuals, and older adults are more prone to urinary incontinence. This is the first study to investigate bacterial species concordance between these two anatomical sites. We found, using both culture-dependent and -independent methods, that the same bacterial species can colonize both the urine and wound in one patient at one point in time. Further studies are needed to investigate if these species are of the same lineage and if the urinary microbiota are able to seed colonization of these types of open wounds.

## INTRODUCTION

Chronic open nonhealing wounds severely impact a person’s quality of life and are estimated to impose $25 billion on the health care system annually within the United States (1–3). Older adults are at greater risk of developing chronic open nonhealing wounds, which can include pressure injuries (previously known as pressure ulcers), diabetic foot ulcers, arterial insufficiency ulcers, and venous leg ulcers (4–6). Pressure injuries in nursing home residents can occur related to skin breakdown over bony prominences related to immobility. It is important to focus on prevention of chronic wounds in this population, as the rate of wound repair and healing reduces with increased age (5). Additionally, bacterial colonization in open wounds can contribute to impaired wound healing (7, 8). Open pressure injuries in nursing home residents are susceptible to contamination of microorganisms from the environment, which includes caregivers. They are also susceptible to contamination by indigenous skin microorganisms, which can invade and become pathogenic once the skin barrier is damaged (9). These factors make it important to focus efforts on preventing bacterial contamination of open wounds. In order to design interventions to prevent wound contamination and invasive infection, it is imperative to understand all sources of possible contamination.

In older adults, urinary incontinence is common (8, 10, 11). When all other types of management and treatments for urinary incontinence fail to keep the skin dry, bladder catheterization is often performed. This is particularly important when the patient has an open wound in a body location, such as the sacrum, that is challenging to keep clear of urine or feces (10, 12–15). However, it is well known that indwelling urinary catheters can lead to increased bacterial colonization, including colonization by antibiotic-resistant organisms, and can promote urinary tract infection (14, 16, 17). Within nursing home residents, urinary tract infections are the most common type of infection (18, 19). To prevent urinary tract infections, multiple recommendations regarding best approaches to catheter care have been made, including the reduced use of catheters in this patient population (20–23). Recent studies confirm the presence of a resident microbial community in urine from both catheterized and noncatheterized patients (24–26); however, the impact of these communities on wound contamination remains unknown.

In this pilot study, we sought to determine if bacterial species found in urine of nursing home residents would also be found within open wounds in the same patient at the same point in time. We hypothesized that open wounds located between the umbilicus and mid-thigh could harbor bacterial species also found in the nursing home resident’s urine. To address this question, we identified bacterial species from urine and open wounds using both culture-dependent and -independent methods.

## RESULTS

### Characteristics of participants involved in this pilot study.

In this pilot study, a total of 13 residents from two different nursing home facilities were recruited; however, samples from only 9 residents were included in this study (Table 1). Four residents and their samples were excluded from this analysis for not having concurrent urine and wound samples. The mean age for residents included in this analysis was 70 years, and all residents were male and Caucasian. Residents enrolled in this study remained in their respective nursing homes for a mean of 131 days, with a range of 10 to 740 days. Most residents in this study were described as immobile (*n* = 8 [89%]). Of those 8 residents, immobility was described to be resulted from paraplegia (*n* = 4), morbid obesity (*n* = 2), and unknown (*n* = 2). A proportion of residents in this study were documented as having urinary incontinence (*n* = 4 [44%]), two of which also had fecal incontinence. Each resident, with the exception of resident 8, had only one eligible open wound in the area of interest. Resident 8 had two eligible open wounds in the area of interest.

**TABLE 1:** Participant characteristics at first sampling time point

Characteristic	Result for residents (*n* = 9)
Demographic	
Age, mean yr	70
Male, no. (%)	9 (100)
Race, no. (%) Caucasian	9 (100)
Length of stay, mean days (range)	131 (10–740)

Mobility, no. (%)	
Immobile	8 (89)
Paraplegia	4 (44)
Morbid obesity	2 (22)
Etiology not further specified	2 (22)

Incontinence, no. (%)	
Total	4 (44)
Fecal and urinary	2 (22)
Urinary only	2 (22)

Method for urine specimen collection, no. (%)	
Clean catch (no catheter use)	3 (33)
Intermittent straight catheter (ISC)	4 (44)
Indwelling transurethral (Foley) catheter	2 (22)

### Microbial community structure of urine and open wounds.

A total of 15 concurrent urine and wound samplings from 9 patients were included in the analysis, with 6 subjects sampled at only 1 time point and 3 subjects sampled at multiple time points. To obtain a global view of bacteria present in urine and wound from these residents, we used culture-independent analysis that involved sequence analysis of V4 region amplicons of the 16S rRNA gene. This method has been used to characterize the community structure of complex microbial consortia from a variety of habitats, including the urogenital tract. A total of 1,411,374 sequences were generated in this data set, with an average of 45,528 sequences per sample (a minimum of 7,395 and maximum of 141,600 sequences per sample). An extraction control and two PCR-negative controls were processed, with an average of 442 reads per sample (range, 15 to 782). For urine samples, an average of 47,310 reads were generated per sample (range, 11,817 to 102,831), whereas open wound samples had an average of 43,627 reads per sample (range, 7,395 to 141,600). Samples with less than 7,395 reads were removed from this data set. Out of 34 samples, 4 were excluded from this data set for having fewer than 7,395 reads.

**FIG 1:**
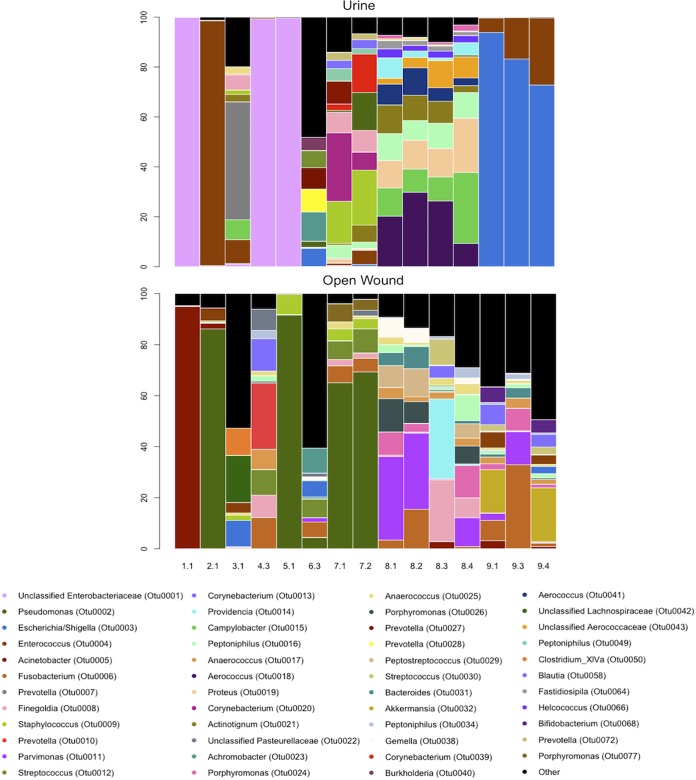
Microbial community structure of urine and open wounds in this study. The microbial community structure of urine and open wounds was determined through amplification of the V4 16S rRNA gene followed by Illumina sequencing. Sequence data were processed and analyzed using the software package mothur. Samples were normalized to 7,395 sequences per sample, and the bar plot was visualized using R. In some cases, urine samples were dominated by one OTU, whereas wound samples were more frequently found to be characterized by many species. The *x* axis identifies the resident and sampling time point (e.g., 1.1 for resident 1 at the first sampling time point). The *y* axis represents the relative abundance of each OTU.

This analysis revealed the presence of a multispecies bacterial community in most samples (Fig. 1). In general, a greater diversity of organisms was present in wound samples compared to urine samples. Urine samples had a mean observed species richness of 44.6 (minimum, 5; maximum, 151; standard deviation, 39.96), compared to an average of 74.5 for wound samples (minimum, 19; maximum, 164; standard deviation, 49) (see Table S1 in the supplemental material). Residents varied with respect to the nature of the community structure in both urine and open wounds. In residents who had more than one sample obtained over time, the communities at one site were more similar over time than the communities in other residents (Fig. 1).

10.1128/mSphere.00463-19.2 TABLE S1 Richness and evenness of urine and open wound samples. Download Table S1, PDF file, 0.02 MB. Copyright © 2019 Libertucci et al. 2019 Libertucci et al. This content is distributed under the terms of the Creative Commons Attribution 4.0 International license.

In addition to the greater diversity observed in wound samples compared to urine, urine was more likely to be characterized by the presence of one dominant operational taxonomic unit (OTU) compared to wounds (Fig. 1). For example, OTU0001 (unclassified *Enterobacteriaceae*) was the only type of bacterium seen in urine samples from residents 1, 4, and 5, while the three urine samples from resident 9 were dominated by OTU0003 (*Escherichia*/*Shigella*). In wound samples, greater diversity was observed, although a minority of communities had one dominant OTU. For example, resident 1 had an overabundance of OTU0005 (Acinetobacter), whereas residents 2, 5, and 7 had an overabundance of OTU0002 (*Pseudomonas*).

### Culture-based analysis of urine and wound specimens.

For clinical purposes, bacterial culture has been the mainstay of microbial isolation and identification. We isolated bacteria from urine and open wounds using aerobic culture on standard microbiologic media and identified organisms obtained through the use of matrix-assisted laser desorption ionization–time of flight mass spectrometry (MALDI-TOF MS) (see Table S2 in the supplemental material). One hundred eighteen bacterial isolates were obtained from 15 wound and 15 urine samples. These 118 isolates were identified as a total of 31 different species. In 12 of 15 urine samples and 15 of 15 wound samples, more than one species was identified by culture. Within urine samples, the most frequently isolated bacteria were Enterococcus faecalis, Aerococcus sanguinicola, Escherichia coli, Proteus mirabilis, and Providencia stuartii. Within open wounds, *Staphylococcus* and *Corynebacterium* species were commonly isolated from residents, as well as E. faecalis, Pseudomonas aeruginosa, and Acinetobacter baumannii.

10.1128/mSphere.00463-19.3 TABLE S2 Comparison of MALDI-TOF, 16S Sanger sequencing, and 16S rRNA-encoding gene amplicon sequencing. Download Table S2, XLSX file, 0.01 MB. Copyright © 2019 Libertucci et al. 2019 Libertucci et al. This content is distributed under the terms of the Creative Commons Attribution 4.0 International license.

To compare the results of cultivation to the culture-independent analysis, partial 16S rRNA-encoding gene sequences were obtained from each cultivar. These partial 16S sequences were compared to the community 16S data to assign each cultivar to an OTU identified by the culture-independent analysis. With two exceptions (both were cultivars identified as Corynebacterium aurimucosum by MALDI-TOF MS), all of the cultivars mapped to an OTU identified by community analysis (Table S2). For 6 of 15 urine samples, the cultivated organisms were the most abundant OTUs encountered by 16S rRNA gene sequencing. This was comparable to open wound samples, where for 5 out of 15 samples, the cultivar was the most abundant OTU (Table 2).

**TABLE 2:** Relative abundance of cultivar identified OTUs within each sample

Time point	Urine			Wound		
	Most abundant OTU in sample (%)^*a*^	MALDI-TOF ID of cultivar corresponding to most abundant OTU	% of community represented by all cultivars	Most abundant OTU in sample (%)^*a*^	MALDI-TOF ID of cultivar corresponding to most abundant OTU	% of community represented by all cultivars
1.1	OTU0001, *Enterobacteriaceae* unclassified (99.9)	*Enterobacter asburiae*	99.99	OTU0005, *Actinobacter* (95)	Acinetobacter baumannii	95.2
2.1	OTU0004, *Enterococcus* (98.0)	*Enterococcus faecalis*	98.1	OTU0002, *Pseudomonas* (86.0)	*Pseudomonas aeruginosa*	91.7
3.1	OTU0007, *Prevotella* (47.2)	Not captured by cultivation	10.8	OTU0042, *Lachnospiraceae* unclassified (18.5)	Not captured by cultivation	19.9
4.3	OTU0001, *Enterobacteriaceae* unclassified (99.3)	*Klebsiella pneumoniae*	99.9	OTU0010, *Prevotella* (26.0)	Not captured by cultivation	0
5.1	OTU0001, *Enterobacteriaceae* unclassified (99.7)	*Citrobacter amalonaticus*	99.7	OTU0002, *Pseudomonas* (92.0)	*Pseudomonas aeruginosa*	99.5
6.3	OTU0023, *Achromobacter* (12)	Not captured by cultivation	0.2	OTU0023, *Achromobacter* (10.0)	Not captured by cultivation	4.3
7.1	OTU0020, *Corynebacterium* (27.3)	*Corynebacterium aurimucosum*	31.9	OTU0002, *Pseudomonas* (65.0)	*Pseudomonas aeruginosa*	77.0
7.2	OTU0009, *Staphylococcus* (22.0)	*Staphylococcus capitis*	46.6	OTU0002, *Pseudomonas* (69.2)	*Pseudomonas aeruginosa*	73.2
8.1	OTU0018, *Aerococcus* (20.2)	*Aerococcus sanguinicola*	47.6	OTU0011, *Parvimonas* (32.7)	Not captured by cultivation	1.0
8.2	OTU0018, *Aerococcus* (30)	*Aerococcus sanguinicola*	55.0	OTU0011, *Parvimonas* (30.0)	Not captured by cultivation	1.7
8.3	OTU0018, *Aerococcus* (26.2)	*Aerococcus sanguinicola*	38.7	OTU0014, *Providencia* (31.3)	Providencia stuartii	52.8
8.4	OTU0015, *Campylobacter* (28.4)	Not captured by cultivation	36.7	OTU0032, *Akkermansia* (21.1)	Not captured by cultivation	2.8
9.1	OTU0003, *Escherichia*/*Shigella* (93.8)	Escherichia coli	99.8	OTU0032, *Akkermansia* (16.9)	Not captured by cultivation	11.2
9.3	OTU0003, *Escherichia*/*Shigella* (93.8)	Escherichia coli	99.7	OTU0006, *Fusobacterium* (32.8)	Not captured by cultivation	1.7
9.4	OTU0003, *Escherichia*/*Shigella* (72.8)	Escherichia coli	99.7	OTU0002, *Pseudomonas* (91.6)	*Pseudomonas aeruginosa*	92.3

a Culture-independent analysis.

### Bacteria detected in both urine and open wounds per resident.

We used both culture-dependent and -independent methods to identify the presence of bacterial species within both urine and open wounds obtained at the same time (Table 3). Using bacterial culture, at least one bacterial species was detected in both urine and an open wound for 13 out of 15 time points. In two cases, all bacterial species that were detected in urine were detected within a resident’s open wound (resident 3 at sampling time point 1 and resident 7 at sampling time point 2). Using culture-independent characterization of the wound and urine microbiota, at least 1 OTU was shared by both urine and open wounds for all 15 sampling time points.

**TABLE 3:** Number of bacterial species or OTUs detected both in urine and open wounds at the same sampling time point per resident

Resident	Sampling time point	Culture dependent			Culture independent		
		No. of unique species detected in:		No. of bacterial species found in both urine and wound	No. of unique OTUs detected in:		No. of OTUs found in both urine and wound
		Urine	Open wound		Urine	Open wound	
1	1	1	2	0	5	32	1
2	1	1	3	1	26	43	8
3	1	3	3	3	73	79	50
4	3	2	4	1	9	38	1
5	1	3	4	2	5	19	2
6	3	1	2	0	151	163	106
7	1	5	4	1	57	31	13
	2	4	4	2	51	29	9
8	1	4	9	2	63	56	19
	2	4	10	2	65	61	19
	3	3	6	2	71	75	20
	4	4	14	3	62	71	19
9	1	2	5	2	11	142	5
	3	2	5	2	10	115	4
	4	2	7	2	10	164	4

Of the 30 bacterial species isolated in at least one sample of urine or wound using culture-based analysis, there were 6 bacterial species isolated from both urine and an open wound at the same sampling time point for a resident (Table 4). This included (in descending order of frequency) E. faecalis, P. mirabilis, E. coli, P. stuartii, P. aeruginosa, and Citrobacter farmeri. Using culture-independent methods (see Table S3 in the supplemental material), the community analysis data revealed that 164 OTUs were shared between urine and open wounds. The OTUs that were most frequently found in both the urine and wound at the same sampling time point were OTU0003 (*Escherichia*/*Shigella*), OTU0004 (*Enterococcus*), OTU0031 (*Bacteroides*), OTU0017 (*Anaerococcus*), OTU0021 (*Actinotignum*), and OTU0025 (*Anaerococcus*).

**TABLE 4:** Frequency of bacterial species by culture-dependent identification in urine, open wound, and both urine and wound

Cultivar	No. of times species detected in:		Frequency detected in both urine and wound at same sampling time point
	Urine (*n* = 15 time points)	Open wound (*n* = 15 time points)	
Enterococcus faecalis	9	13	9 (9/15 [60%])
Proteus mirabilis	5	7	4 (4/15 [27%])
Escherichia coli	4	4	4 (4/15 [27%])
Providencia stuartii	4	4	4 (4/15 [27%])
Pseudomonas aeruginosa	3	7	3 (3/15 [20%])
Citrobacter farmeri	1	1	1 (1/15 [7%])
Aerococcus sanguinicola	4	0	0 (0/15 [0%])
Aerococcus urinae	2	0	0 (0/15 [0%])
Corynebacterium aurimucosum	2	0	0 (0/15 [0%])
Acinetobacter baumannii	1	8	0 (0/15 [0%])
Staphylococcus aureus	1	5	0 (0/15 [0%])
Staphylococcus epidermidis	1	2	0 (0/15 [0%])
Citrobacter amalonaticus	1	0	0 (0/15 [0%])
Enterobacter asburiae	1	0	0 (0/15 [0%])
Klebsiella pneumoniae	1	0	0 (0/15 [0%])
Staphylococcus capitis	1	0	0 (0/15 [0%])
Corynebacterium striatum	0	7	0 (0/15 [0%])
Morganella morganii	0	3	0 (0/15 [0%])
Staphylococcus cohnii	0	3	0 (0/15 [0%])
Staphylococcus simulans	0	3	0 (0/15 [0%])
Arthrobacter cumminsii	0	2	0 (0/15 [0%])
Corynebacterium amycolatum	0	2	0 (0/15 [0%])
Streptococcus agalactiae	0	2	0 (0/15 [0%])
Brevibacterium ravenspurgense	0	1	0 (0/15 [0%])
Enterococcus avium	0	1	0 (0/15 [0%])
Globicatella sanguinis	0	1	0 (0/15 [0%])
Providencia rettgeri	0	1	0 (0/15 [0%])
Serratia marcescens	0	1	0 (0/15 [0%])
Streptococcus constellatus	0	1	0 (0/15 [0%])
Streptococcus mitis	0	1	0 (0/15 [0%])

10.1128/mSphere.00463-19.4 TABLE S3 OTUs found in both urine and wounds in one patient at one point in time. Download Table S3, XLSX file, 0.01 MB. Copyright © 2019 Libertucci et al. 2019 Libertucci et al. This content is distributed under the terms of the Creative Commons Attribution 4.0 International license.

## DISCUSSION

In this pilot study, we assessed how often the same bacterial species was identified in both urine and open wounds from nursing home residents for specimens collected at the same sampling time point. This was assessed for wounds located in body areas anticipated to be at high risk of contamination from urine. Comparison of bacterial species identified from different anatomical sources in the same patient has been evaluated previously in an effort to identify possible sources of bacteremia (27), noting some organisms responsible for bloodstream infections were also found in urine and wound microbiota. Additionally, concordance between urinary tract infections and positive blood cultures in neonates has been previously identified (28). Although it is well recognized that contamination of open wounds by microorganisms can lead to infectious complications such as cellulitis, osteomyelitis, bacteremia, and delayed wound healing (5, 7, 29, 30) and that urine is frequently colonized (i.e., asymptomatic bacteriuria) in nursing home residents (18, 31), we believe this is the first study to identify bacterial species present in both urine and open wounds at the same time point, using both culture-dependent and culture-independent methods.

In this pilot study, we found bacterial species that were present in both urine and open wounds in nursing home residents at a specific time point, using both culture-dependent and -independent techniques. This does not implicate the urine as the ultimate source for open wound contamination in these residents. Further investigations are needed to understand if these bacterial species are of the same lineage to provide evidence that the urine is able to seed open wound colonization. However, our results indicate that there were bacterial species that colonized both urine and open wounds at a greater frequency than other bacterial species in one resident at one point in time (Table 4). E. faecalis, P. mirabilis, E. coli, P. stuartii, and P. aeruginosa were found to most often colonize both the urine and open wound in one resident at one point in time in our cohort, using culture-dependent methods. These bacterial species are not indigenous to the skin (32, 33), but many studies have shown that they are commonly isolated from open wounds (34, 35) and from urinary tract infections (35, 36). However, it remains unclear if urinary colonization of these microorganisms increases the risk of open wound colonization below the umbilicus. These findings indicate that further study of the relationship between microbes in the urine and on open wounds may help define the precise risk that bacterial colonization of the urine poses for contamination of open wounds.

We initially performed this study by employing standard microbiologic culture to isolate organisms and MALDI-TOF MS to identify those organisms from both urine and open wounds. A comparison between MALDI-TOF MS and 16S Sanger sequencing revealed an almost perfect concordance between the two methods. However, a comparison between culture-dependent and -independent methods in this study did not yield the same concordance as Sanger sequencing and MALDI-TOF MS did. The culturing methods used in this study are widely adopted and commonly used in the clinical setting to detect urine and wound pathogens (37–39). However, standard culture methods used for diagnosis and clinical treatment are designed to selectively enrich for target organisms. A 2016 study has found that these standard culturing conditions often miss non-E. coli uropathogens, including *Klebsiella* and *Proteus* species (40). To address these issues, enhanced culturing methods have been created and validated (25, 40–42), but these culturing methods are not commonly used within the clinical setting. Since standard clinical culture methods are designed to selectively enrich for target organisms, we sought to determine if the use of a more comprehensive method to retrieve information on the potential bacterial communities in urine and wounds would provide additional insight into the microbiota in these two anatomic sites. As expected, profiling the bacteria in urine and wounds using 16S rRNA-encoding gene sequence analysis provided evidence for a greater diversity of organisms than that encountered using culture alone. Using 16S rRNA-encoding gene sequencing to detect the same OTU in a resident found shared OTUs between both sites at all sampling time points. Similarly, to our culture results, there were some OTUs that were more frequently detected in both the urine and wound in one resident at one point in time. Those OTUs that were most commonly concordant between both sites in a resident at one point in time were not found within our extraction or PCR controls, except for OTU0003, which was found in small amounts, providing confidence that those OTUs were not a result of contamination from processing these samples. However, whether or not those OTUs were viable can only be determined through culturing, highlighting the importance of culturing anaerobes at both of these anatomical sites. Supporting the notion of the cultivation of anaerobes at these two anatomical sites, we showed that in some cases, culture-independent analyses identified anaerobes as the most abundant OTUs identified in a given sample. This has been noted in previous studies that identified the presence of 16S genes from anaerobes in chronic wounds and urine (43, 44). Admittedly, 16S amplicon analysis cannot provide high-confidence evidence that phylotypes found in the two locations in the same patient were transmitted from one site to the other. This would require a finer-scale technique such as whole-genome sequencing, which was beyond the scope of this pilot investigation.

Overall, this pilot study indicates that bacterial species from urine can often be found in open wounds within the same patient at a point in time in nursing home residents, with or without urinary catheter use, using both culture-dependent and -independent methods. This study warrants further investigation into the role of urinary microorganisms within open wound contamination. Another important consideration is that some species are found at higher frequency than others, which suggest that they are more robust and can live in many environments. This is of particular concern as many of these organisms can often be found to be resistant to antibiotics and in some cases are resistant to multiple types of antibiotics. Further investigation is needed to fully understand the relationship and clinical implications of organisms found in both urine and open wounds in the nursing home population.

## MATERIALS AND METHODS

### Study design and participants.

This study was approved by the institutional review board at the University of Michigan (HUM00092777). All participants (or approved decision makers) provided written informed consent prior to the initiation of this investigation. Subjects enrolled in this study were nursing home residents recruited from two nursing homes located in southeast Michigan. Recruitment for nursing home A occurred between March 2015 and May 2016, whereas recruitment from nursing home B occurred between January 2016 and May 2016. Both facilities included short- and long-term care beds. In order for residents to be eligible for this study, they had to be a minimum of 18 years of age and have an open wound in the anatomical area of interest (between the umbilicus and mid-thigh). For the purposes of this investigation, open wounds included pressure injuries (at stages 2 to 4) and other open wounds with potential multiple etiologies (e.g., burns or surgical incisions) that were not anticipated to have a direct connection with the gastrointestinal or genitourinary tract. Nursing home residents with a closed wound (such as a stage 1 pressure injury) or wounds expected to have a connection with the gastrointestinal tract and/or urinary tract, such as perirectal, fistula, and ostomies, were ineligible for this study. Additional criteria that deemed a resident ineligible to enroll in this study included residents who were receiving end-of-life care or were anticipated to be discharged prior to the completion of the study, the presence of nephrostomy tube or ileal conduit urinary diversion as a primary means of urine output, or if the resident was anuric. Participants were subject to withdrawal from this study if their open wound healed, the patient was discharged or admitted to the hospital, or the patient retracted consent prior to the completion of sample collection. Patient data were collected by L.M., J.M., and B.L. and included demographics, reason for admission, wound and catheter (if applicable) information, continence (fecal and urinary), functional status, mobility, relevant medical history, and comorbidities.

### Specimen collection.

All wound swabs were collected during regularly scheduled wound dressing changes by nursing staff in order to minimize disturbance to the healing process. Some subjects were sampled at multiple time points. At each sampling time point, two wound swabs were taken using the Levine method (45). Briefly, wound swabs were taken by twirling the end of a sterile cotton-tipped applicator stick on the open wound in a 1-cm^2^ area for 5 s. Two types of cotton swabs were used to collect samples from open wounds: BactiSwab (Starplex Scientific, Inc., Ontario, Canada) and BactiSwab Dry (Remel, Lenexa, KS). BactiSwab was used to collect samples for downstream culture-dependent identification, and BactiSwab Dry was used to collect samples for downstream culture-independent identification. Urine specimens were collected from residents at the same sampling time point as wound swabs were collected. Urine specimens were collected by midstream void (in residents able to void without a urinary catheter) or by aseptic collection from urinary catheter and aliquoted for culture-dependent and -independent identification. The order of sampling these sites was determined by the availability of the wound nursing team. This meant that sometimes the urine was sampled before the wound and vice versa.

### Culture isolation and identification of bacterial species by MALDI-TOF MS analysis.

Bacteria were isolated from wound swabs and urine samples by streaking specimens onto Trypticase soy agar with 5% sheep blood (PA-254053.07; Becton Dickinson, Franklin Lakes, NJ), Columbia agar with 5% sheep blood (PA-254005.06; Becton Dickinson, Franklin Lakes, NJ), MacConkey agar (L007388; Becton Dickinson, Franklin Lakes, NJ), and bile esculin agar (dehydrated [299068; Becton Dickinson, Franklin Lakes, NJ]). For urine samples, 10 μl of the sample was plated. Plates were incubated at 35°C for 24 h. Colonies with distinct morphology were picked and grown in 5 ml of brain heart infusion (BHI) medium for 18 h, shaking at 37°C (dehydrated DF0418-17-7; Becton Dickinson, Franklin Lakes, NJ). Freezer stocks were made by mixing 500 μl of liquid culture with 500 μl of glycerol (BP229-1; Fisher BioReagents, Pittsburgh, PA) and stored at –80°C. Bacterial species were identified by streaking each freezer stock onto BHI agar (241830; Becton Dickinson, Franklin Lakes, NJ) using a 1-μl disposable inoculating loop (22-031-21; Fisher Scientific, Hampton, NH) and grown at 37°C for 24 h. Colonies were identified using MALDI-TOF analysis on a Bruker MALDI Biotyper CA system. A single colony was smeared, using the blunt end of a wooden toothpick, onto a reusable polished steel target plate (MSP 96 target polished steel BC 8280800; Bruker Daltonik, Bremen, Germany). For each MALDI-TOF run, two positive controls were added to the target plate, which included a Gram-negative control (Escherichia coli ATCC 25922) and a Gram-positive control (Staphylococcus aureus ATCC 25923). The target plate was air dried prior to the addition of a matrix solution and formic acid using the MALDI Biotyper Galaxy automated target plate preparation system (1836007; Bruker Daltonik, Bremen, Germany). Analysis was performed as per the manufacturer’s instructions. Bacterial identification was taken from the identified best match with a score of ≥2.00.

### Identification of bacterial cultivars using Sanger sequence analysis of the 16S rRNA-encoding gene.

Bacterial isolates were streaked onto BHI agar, from each freezer stock, using a 1-μl disposable inoculation loop and grown at 37°C for 24 h. A single colony was grown in 5 ml of BHI medium at 37°C for 18 h with shaking. Bacteria were then harvested by centrifugation of 1 ml of culture for 1 min at 10,000 rpm. Harvested pellets were added to a Masterblock 96-deep-well microplate (780271; VWR, Radnor, PA) for genomic DNA extraction using the PowerMag Soil DNA isolation kit (Mo Bio Laboratories, Inc., Carlsbad, CA). Automated isolation was performed using an EpMotion 5075 instrument (Eppendorf, Hamburg, Germany). In some cases, genomic DNA was isolated by hand using the DNeasy blood and tissue kit (69504; Qiagen, Venlo, Limburg, Netherlands). Molecular identification was completed by sequence analysis of the 16S rRNA-encoding gene. One microliter of extracted genomic DNA (1 to 20 ng/μl) was amplified in a 20-μl reaction mixture that contained: 11.85 μl of ultrapure DNase/RNase-free distilled water (10977023; Invitrogen, Waltham, MA), 2 μl of 10× AccuPrime PCR buffer II (12346-086; Invitrogen, Waltham, MA), 4 μM forward primer (27F, 5′ AGA GTT TGA TCM TGG CTC AG 3′), 4 μM reverse primer (1492R, 5′ GGT TAC CTT GTT ACG ACT T 3′), and 0.15 μl of *Taq* DNA polymerase (2346-086; Invitrogen, Waltham, MA). PCR amplification was performed under the following conditions: an initial denaturation step for 2 min at 95°C followed by 30 cycles of denaturation (95°C for 20 s), primer annealing (55°C for 15 s), and extension (72°C for 5 min), followed by a final extension step (72°C for 10 min). Amplicons were prepared for sequencing by adding 0.25 μl of ExoSAP-IT (78250; Affymetrix, Santa Clara, CA) to 3.25 μl of ultrapure DNase/RNase-free distilled water and 1.5 μl of amplicon and incubated for 30 min at 37°C, followed by 15 min at 80°C. Cleaned PCR products were sent for Sanger sequencing at the University of Michigan Medical School DNA Sequencing Core using nested 16S rRNA gene primers (338F, 5′ ACT CCT ACG GGA GGC AGC 3′; and 907R, 5′ CCG TCA ATT CMT TTG AGT TT 3′). Identification was performed using the Ribosomal Database Project (RDP) classifier (release 11, update 5, 30 September 2016) (46).

### Urine and wound bacterial community analysis via 16S rRNA gene amplicon sequence analysis.

For urine, 1 ml of the specimen was added to a glass bead tube (26000-50-BT; Mo Bio Laboratories, Inc., Carlsbad, CA) and centrifuged for 10 min at 5,000 × *g*. The supernatant was discarded, and this process was repeated using an additional 1 ml of urine. The urine pellets were then stored at –80°C until processed for DNA extraction. For wound samples, the tip of the wound swab was cut, placed into microcentrifuge tubes, and stored at –80°C until processed. Genomic DNA from urine and wound swabs were extracted using the MagAttract PowerMicrobiome DNA/RNA EP kit (formerly known as the PowerMag Microbiome RNA/DNA isolation kit, 27500-4-EP; Mo Bio Laboratories, Inc., Carlsbad, CA) using the Eppendorf EpMotion liquid handling system.

The 16S rRNA-encoding gene from genomic DNA was amplified using barcoded dual-index primers developed by Kozich et al. (47). The process used for library generation was previously described by Seekatz et al. (48). Following amplification, the DNA amplicons in each PCR were normalized using the SequalPrep normalization plate kit (Thermo Fisher Scientific, catalog no. A1051001). The normalized reaction mixtures were pooled and quantified using the Kapa Biosystems Library quantitative PCR (qPCR) MasterMix (ROX Low) quantification kit for Illumina platforms (catalog no. KK4873). The pooled amplicon library was sequenced on the Illumina MiSeq platform using the 500-cycle MiSeq V2 reagent kit (catalog no. MS-102-2003) according to the manufacturer’s instructions, with modifications of the primer set with custom read 1/read 2 and index primers added to the reagent cartridge (47). Libraries with a final load concentration of 5.5 pM, spiked with 15% PhiX, were used (47). DNA isolation and community sequencing were done by the University of Michigan Microbial Systems Molecular Biology Laboratory.

Sequence data were processed and analyzed using the software package mothur (v.1.39.5) and the MiSeq SOP (accessed on 20 November 2018) (47). Sequences were aligned to a recreated V4-specific SILVA SEED reference (release 132) and trimmed (49). Chimeras were then removed using uchime (50). Operational taxonomic units (OTUs) were created using the OptiClust algorithm, which is a *de novo* approach (51). For downstream applications, samples with less than 7,395 sequences per sample were removed from this data set. OTU concordance between each urine and open wound sample per time point per resident was calculated using the mothur calculator sharedsobs. See Text S1 in the supplemental material for the line-by-line code used to process these sequences.

10.1128/mSphere.00463-19.1 TEXT S1 mothur batch file. Download Text S1, PDF file, 0.02 MB. Copyright © 2019 Libertucci et al. 2019 Libertucci et al. This content is distributed under the terms of the Creative Commons Attribution 4.0 International license.

To identify the OTU classification for each bacterial cultivar, the following steps were taken. First, 16S sequences were generated using Sanger sequencing as described in the section “Identification of bacterial cultivars using Sanger sequence analysis of the 16S rRNA-encoding gene.” Then each sequence was aligned to the recreated V4-specific SILVA SEED reference created for the microbiome analysis as described. Once the V4 sequence for each cultivar was identified, those partial sequences were aligned to the created OTUs (as described above). The output was matched to specific OTUs from the community analysis.

### 16S rRNA gene amplicon sequencing controls.

In order to differentiate between artifacts, contaminants, and the present microbial community, a genomic DNA extraction control and PCR-negative controls were processed with patient samples. A mock community, to help identify false-positive reads, was processed as well. We used the ZymoBIOMICS microbial community standard (D6300; ZymoBIOMICS, Irvine, CA), whose composition has been validated to assess bias in DNA isolation. A fasta file of the mock community used in this study can be downloaded at https://microbe.med.umich.edu/sites/default/files/downloads/zymo.mock_.16S_fasta.txt.

### Availability of data.

Raw FASTQ files, including those for negative controls and the mock community, have been deposited in the SRA database under BioProject ID no. PRJNA533783. Detailed processing steps can be found in Text S1 in the supplemental material.
