# Supplementary material for: Bacteria Detected in both Urine and Open Wounds in Nursing Home Residents: a Pilot Study
Source: mSphere. 2019 Aug 28;4(4):e00463-19. doi: 10.1128/mSphere.00463-19 (PMC6714893; doi:10.1128/mSphere.00463-19)
Supplement: TABLE S1 [file mSphere.00463-19-st001.pdf]

Supplemental Table 1: Richness and evenness of urine and open wound samples

		Urine		Open Wound	
Resident	Sampling timepoint	Species Observed	Richness and Evenness (Shannon)	Species Observed	Richness and Evenness (Shannon)
1	1	5	0.007593	32	0.325857
2	1	26	0.146862	43	0.745223
3	1	73	2.201193	79	2.956122
4	3	9	0.044153	38	2.331146
5	1	5	0.024944	19	0.319696
6	3	151	3.415258	163	3.408012
7	1	57	2.58421	31	31
	2	51	2.494866	29	1.239733
8	1	63	2.51202	56	2.398028
	2	65	2.359984	61	2.426502
	3	71	2.481279	75	2.383013
	4	62	2.217427	71	2.987491
9	1	11	0.246021	142	3.567696
	3	10	0.465196	115	2.767058
	4	10	0.607312	164	3.552591
Mean		44.6	1.45	74.53	4.16
Range [min – max]		5 - 151	0.0076 – 3.42	19 - 164	0.32 - 31
Standard Deviation		39.96	1.23	49.11	7.5
