# Supplementary material for: Bacteria Detected in both Urine and Open Wounds in Nursing Home Residents: a Pilot Study
Source: mSphere. 2019 Aug 28;4(4):e00463-19. doi: 10.1128/mSphere.00463-19 (PMC6714893; doi:10.1128/mSphere.00463-19)
Supplement: TEXT S1 [file mSphere.00463-19-s0001.pdf]

```
##Meddings_Pepper_Microbial_concordance
##November 20, 2018
##Josie Libertucci
##Mothur v.1.39.5

#Before starting this batch in mothur, unzipped fastq files:
#gunzip *.fastq.gz

make.contigs(file=meddings_forpub.files, processors=2)
summary.seqs(fasta=meddings_forpub.trim.contigs.fasta, processors=2)
screen.seqs(fasta=meddings_forpub.trim.contigs.fasta,
group=meddings_forpub.contigs.groups, maxambig=0, maxlength=275,
processors=2)
unique.seqs(fasta=meddings_forpub.trim.contigs.good.fasta)
count.seqs(name=meddings_forpub.trim.contigs.good.names,
group=meddings_forpub.contigs.good.groups)
summary.seqs(count=meddings_forpub.trim.contigs.good.count_table,
processors=2)
pcr.seqs(fasta=silva.seed_v132.align, start=11894, end=25319,
keepdots=F, processors=2)
system(mv silva.seed_v132.pcr.align silva.v4.fasta)
summary.seqs(fasta=silva.v4.fasta, processors=2)
align.seqs(fasta=meddings_forpub.trim.contigs.good.unique.fasta,
reference=silva.v4.fasta, processors=2)
summary.seqs(fasta=meddings_forpub.trim.contigs.good.unique.align,
count=meddings_forpub.trim.contigs.good.count_table, processors=2)
screen.seqs(fasta=meddings_forpub.trim.contigs.good.unique.align,
count=meddings_forpub.trim.contigs.good.count_table,
summary=meddings_forpub.trim.contigs.good.unique.summary, start=1968,
end=11550, maxhomop=8, processors=2)
summary.seqs(fasta=current, count=current, processors=2)
filter.seqs(fasta=meddings_forpub.trim.contigs.good.unique.good.align,
vertical=T, trump=., processors=2)
unique.seqs(fasta=meddings_forpub.trim.contigs.good.unique.good.filter
.fasta, count=meddings_forpub.trim.contigs.good.good.count_table)
pre.cluster(fasta=meddings_forpub.trim.contigs.good.unique.good.filter
.unique.fasta,
count=meddings_forpub.trim.contigs.good.unique.good.filter.count_table
, diffs=2, processors=2)
chimera.uchime(fasta=meddings_forpub.trim.contigs.good.unique.good.fil
ter.unique.precluster.fasta,
count=meddings_forpub.trim.contigs.good.unique.good.filter.unique.prec
luster.count_table, dereplicate=t, processors=2)
remove.seqs(fasta=meddings_forpub.trim.contigs.good.unique.good.filter
.unique.precluster.fasta,
accnos=meddings_forpub.trim.contigs.good.unique.good.filter.unique.pre
cluster.denovo.uchime.accnos)
summary.seqs(fasta=current, count=current, processors=2)
classify.seqs(fasta=meddings_forpub.trim.contigs.good.unique.good.filt
er.unique.precluster.pick.fasta,
```

```
count=meddings_forpub.trim.contigs.good.unique.good.filter.unique.prec
luster.denovo.uchime.pick.count_table,
reference=trainset16_022016.rdp.fasta,
taxonomy=trainset16_022016.rdp.tax, cutoff=80)
remove.lineage(fasta=meddings_forpub.trim.contigs.good.unique.good.fil
ter.unique.precluster.pick.fasta,
count=meddings_forpub.trim.contigs.good.unique.good.filter.unique.prec
luster.denovo.uchime.pick.count_table,
taxonomy=meddings_forpub.trim.contigs.good.unique.good.filter.unique.p
recluster.pick.rdp.wang.taxonomy, taxon=Chloroplast-Mitochondria-
unknown-Archaea-Eukaryota)
count.seqs(name=current, group=current)
count.groups(count=meddings_forpub.trim.contigs.good.unique.good.filte
r.unique.precluster.denovo.uchime.pick.pick.count_table)
cluster.split(fasta=meddings_forpub.trim.contigs.good.unique.good.filt
er.unique.precluster.pick.pick.fasta,
count=meddings_forpub.trim.contigs.good.unique.good.filter.unique.prec
luster.denovo.uchime.pick.pick.count_table,
taxonomy=meddings_forpub.trim.contigs.good.unique.good.filter.unique.p
recluster.pick.rdp.wang.pick.taxonomy, splitmethod=classify,
taxlevel=2, cutoff=0.03, processors=1)
system(mv
meddings_forpub.trim.contigs.good.unique.good.filter.unique.precluster
.pick.pick.opti_mcc.unique_list.list meddings_forpub.final.list)
system(mv
meddings_forpub.trim.contigs.good.unique.good.filter.unique.precluster
.pick.pick.fasta meddings_forpub.final.fasta)
system(mv
meddings_forpub.trim.contigs.good.unique.good.filter.unique.precluster
.pick.rdp.wang.pick.taxonomy meddings_forpub.final.taxonomy)
system(mv
meddings_forpub.trim.contigs.good.unique.good.filter.unique.precluster
.denovo.uchime.pick.pick.count_table
meddings_forpub.final.count_table)
system(mkdir single_files)
system(mv *.map single_files)
count.groups(count=meddings_forpub.final.count_table)
make.shared(list=meddings_forpub.final.list,
count=meddings_forpub.final.count_table, label=0.03)
classify.otu(list=meddings_forpub.final.list,
count=meddings_forpub.final.count_table,
taxonomy=meddings_forpub.final.taxonomy, label=0.03)

#prepare the OTU table for downstream applications via normalization
of these data
remove.groups(shared=meddings_forpub.final.shared, groups=CONTROL_S26-
ExtractioncontrolH12_S315-mockA-08282015_S27-mockD100716_S290-
waterA-08282015_S28-waterD100716_S291)#removing the controls
# created a file called meddings_forpub.final.0.03.pick.shared
#going to normalize dataset for downstream applications
```

```
sub.sample(shared=meddings_forpub.final.0.03.pick.shared, size=7395)

#going to calculate observed OTUs in each sample and then compare the
shared OTUs in each sample
summary.single(shared=meddings_forpub.final.0.03.pick.
0.03.subsample.shared, calc=sobs) #only for richness
summary.single(shared=meddings_forpub.final.0.03.pick.
0.03.subsample.shared, calc=sobs-shannon) #for richness and richness
and evenness
summary.shared(shared=meddings_forpub.final.0.03.pick.
0.03.subsample.shared, calc=sharedsobs, all=TRUE)
```
